# Effect of two formulations of recombinant bovine somatotropin on milk production and body condition of cattle under intensive management in Peru

**DOI:** 10.1007/s11250-021-03036-z

**Published:** 2022-02-09

**Authors:** Carlos A. Gómez, Melisa Fernández, Néstor Franco, Rudi Cueva

**Affiliations:** 1 Facultad Zootecnia, Universidad Nacional Agraria La Molina, Av. La Molina s/n, La Molina, Lima, Perú; 2 Unidad de Innovación, Battilana Nutrición SAC, San Borja, Lima, Perú

**Keywords:** Body condition, Cattle, Milk production, Recombinant somatotropin

## Abstract

The effect of recombinant somatotropin (rbST) application in cattle has been demonstrated in temperate climate but very limited studies are available in tropical regions. The objective of this study was to compare the effect of the application of two different formulations of rbST on the milk yield and body condition of dairy cattle in a commercial herd under intensive production in Peru. We evaluated the application of 500 mg of active rbST in a zinc sesame oil (ZSO-rbST; *n* = 44) or vitamin E lecithin (VEL-rbST; *n* = 45) vehicle while control cows (*n* = 42) did not receive any application. The application of rbST was performed by every 14 days for 12 cycles, for a total of 168 days. The application of rbST increased the milk production of primiparous and multiparous cows by 3 and 3.2 kg/day for the VEL-rbST formulation respectively when compared with control cows (*p* < 0.01) and no difference in milk production was observed between the ZSO-rbST formulation and the control group (*p* > 0.05). However, no significant difference on milk production was observed between the rbST formulations evaluated. The effect of rbST per injection cycle indicated differences in milk production and economic return for the 12 cycles between rbST and control in primiparous group, while in multiparous, no differences were found between ZSO-rbST and control (*p* > 0.05), but differences were observed between VEL-rbST and control in 41% of the cycles (*p* < 0.05). No differences in body condition were found between the two rbST formulations and the control group during the evaluation. In conclusion, the application of rbST promoted higher milk production of cattle which had a positive impact on the economic income of the farmer.

## Introduction

Bovine somatotropin (bST), also known as bovine growth hormone, is a natural protein secreted by the anterior pituitary gland (Estrada and Shirley, 1989) and the circulating concentration is positively correlated with milk production and mammary gland development (Fesseha et al. 2019). In lactating cattle, bST regulates the partitioning of nutrients and its application increases milk production due to improvements in the availability and use of nutrients for milk synthesis (Capper et al. 2008). In 1979, recombinant bST (rbST) was developed, a variant approved for application to dairy cows, and its use was approved in the USA in 1993 (Penagos et al. 2014). Since then, numerous studies in temperate zones have shown that rbST increases milk production and feed efficiency in lactating cows as described in the meta-analysis of St-Pierre et al. (2014) although variable magnitude of responses has been reported with cattle treated with rbST (Chalupa et al. 1988; Penagos et al. 2014) for which it has been identified that management factors are important sources of variation (Shibru, 2016).

Using a meta-analysis of 26 studies of the effect of rbST, the administration of rbST to dairy cows increased milk production by 4.0 kg/day without adverse effects on the health of the cows (St-Pierre et al. 2014). However, the response in milk production of primiparous and multiparous cows treated with rbST is differentiated, being lower in primiparous (Leonard et al. 1990; Posada et al. 2008; Mellado et al. 2011; De Morais et al. 2017). This can be attributed to the fact that primiparous cows are still growing and although they have higher levels of endogenous bST than multiparous (McBride et al. 1988), the nutrients are still being distributed to cover growth requirements in addition to the requirements for milk synthesis (Wathes et al. 2007). On the other hand, it is known that nutritional requirements increase due to milk production, especially energy, which leads the cow to use her body reserves to maintain milk production when these are not covered, reducing body condition (NRC 2001). It has been reported that cows treated with rbST do not change their body condition compared with untreated cows (De Morais et al. 2017) while in other studies, cows treated with rbST lose body condition (Remond et al. 1991, Thomas et al. 1991 and Stehr et al. 2001). Loss of body condition or body weight of lactating cows could have negative effect on their milk production (Roche et al. 2007).

The use of rbST has been of limited research for dairy cattle in tropical areas. In Latin America, there are two commercial rbST somatotropin products in use, similar in provision of rbST but different in their vehicle component: sesame oil and zinc (ZSO-rbST) or lecithin and vitamin E (VEL-rbST). However, there is limited information from studies under tropical areas evaluating the effect of different commercial somatotropin formulations on milk production, which is a weakness for impact assessment in comparison to the extensive research carried out in North America. Furthermore negative effects of cold or hot climates have been documented on the rbST response, with periods of heat stress that generate lower responses in milk production than those under moderate climatic conditions (Soliman and El-Barody, 2014). Studies with Holstein cows conducted in Brazil with two different rbST formulations in commercial farms had opposite results in productive performance. Almeida and Viechnieski (2011) reported more milk production (+ 1.6 kg/day) for cows with VEL-rbST than those treated with ZSO-rbST, while De Morais et al. (2017) reported + 1.3 kg/day of milk for cows with Z-rbST than cows with VEL-rbST. Similarly, Vargas et al. (2006) reported that application of VEL-rbST promoted more milk production under conditions of crossed bred cows on pastoral management in Colombia. These differences may be due to various characteristics: environment, management, feeding, and genetic potential of cattle existing throughout Latin America, which makes the results difficult to replicate for all the realities that exist in the region. In Peru, dairy farmers have available two commercial formulations of rbST; however, there is no data locally comparing their effectiveness. This information would be useful for dairy farmers when choosing the rbST formulation that allows greater milk production and ultimately the profitability of their herds.

## Materials and methods

### Study area

The experiment was carried out from June to December 2019 in a commercial dairy considering the code of ethics for scientific research with animals of the Universidad Nacional Agraria La Molina (TR. No 0358-CU-UNALM) and the consent of the dairy owner. The herd is located on the coast of Peru, which 550 lactating cows in a “dry lot” type housing system. The region’s climate is classified as a subtropical desert climate (Holdridge 1987), with an average temperature of 17.9° C ± 3.4 °C and a relative humidity of 89% for the evaluation period.

### Experimental design

#### Study animals

The selection criteria for the animals at in the study were postpartum Holstein cows between 30 and 90 days in lactation, no signs of mastitis (clinical or subclinical), average milk production > 25 kg/day, and body condition > 2.5 on a 5-point scale (Heinrichs et al. 2016). At the beginning of the study, primiparous and multiparous cows had a milk production of 36 and 40.3 kg/day, respectively.

#### Treatments

A total of 131 cows with 87 ± 15 days in milk, primiparous (50.4%) and multiparous (49.6%), were assigned to the application of VEL-rbST (*n* = 45; Boostin-S, LG Life Sciences), application of ZSO-rbST (*n* = 44; Lactotropin Elanco Animal Health), or a control group without application (*n* = 42). For both of the rbST treatments, each cow was injected with 500 mg of active rbST every 14 days for 12 injection cycles, 168 days in total. The same route of application (depression on either side of the tail head) was used, which consisted of subcutaneous injections, alternating the site for each injection. The initial milk production was recorded, which considers an average of the three consecutive days before the first application of rbST. The cows during the whole trial were fed with a total mixed ration (TMR) formulated according to the nutrient requirements for dairy cattle (National Research Council [NRC], 2001) that consisted of a mixture of concentrate and forage (Table 1). Cows of the three treatments were in the same corrals and received their ration ad libitum (55.7 ± 4.0 kg/day) distributed three times a day.

**Table 1:** Ingredients (kg as fed/cow /day) and chemical composition of the total mixed ration supplied to the cows

Ingredient	(kg as fed/cow/day)
Corn forage fresh	42.50
Ground grain corn	5.78
Soybean meal	3.55
Full-fat soybean meal	1.20
Dried distillery grain with soluble (DDGS)	0.67
Rice polishing	0.75
Molasses sugar cane	0.53
Calcium soap	0.18
Urea	0.06
Salt	0.07
Calcium carbonate	0.22
Sodium sesquicarbonate	0.20
Vitamins and minerals	0.04
Virginiamycyn	0.01
Live Yeast	0.01
Mycotoxins sequestering agent	0.02
**Total**	**55.7**
Chemical composition (g per 100 g of DM)	
Crude protein	16.6
Crude fat	5.2
Ash	7.0
Neutral detergent fiber,	36.0

*DM* dry matter

### Experimental procedure

The cows were milked three times a day and the individual production per milking was recorded daily by means of the scale of the milking system. Body condition was assessed in all cows at the beginning of each injection cycle using a qualification table from 1 to 5 (Heinrichs et al. 2016). Samples of the total mixed ration were taken every 8 weeks throughout the study for chemical analysis. Dry matter, crude protein, ash, and crude fat contents were determined according to the methods of the Association Official Analytical Chemist (AOAC, 2005). The determination of neutral detergent fiber was carried out using the filter bag technique (Ankom, 2017) with a fiber analyzer 200, Ankom Technology Corporation Fairport, NY, USA.

### Statistical analysis

Initially, the normality was evaluated by the Shapiro–Wilk test. The data were analyzed through the mixed model with repeated measures using SAS version 8.0 (SAS Institute Inc., 1999). Four analyses were performed to evaluate the effect of the rbST formulations: milk production throughout the study, milk production in the 12 injection cycles, milk production within the 14 days of injection cycle, and body condition. The mixed model for the analysis of milk production throughout the study (12 injection cycles) considered the milk production as dependent variable, the fixed effects were the treatments (control and 2 rbST formulations) and the number of parity (primiparous and multiparous). The average production during the first 3 days before the start of the study was used as covariance and the random effect was the cow. The mixed model for the analysis of milk production in the 12 injection cycles in primiparous and multiparous cow considered the milk production as dependent variable, the fixed effects were the treatments (control and 2 rbST formulations), injection cycle, and its interaction (3 × 12 factorial design). For the analysis of milk production within the 14 days of injection cycle, the injection cycle fixed effect was replaced by day of injection cycle in the mixed model (3 × 14 factorial design). The average production during the first 3 days before the start of the study was used as covariance and the random effect was the cow in both primiparous and multiparous. The model for analysis of body condition was also mixed where the dependent variable milk production was replaced by body condition score. The average body condition score during the 1 week before the start of the study was used as covariance and the random effect was the cow in both primiparous and multiparous. The means of milk production and body condition were compared by Tukey’s test at a level of significance of 5%.

## Results

### Effect of rbST on milk production

**Fig. 1:**
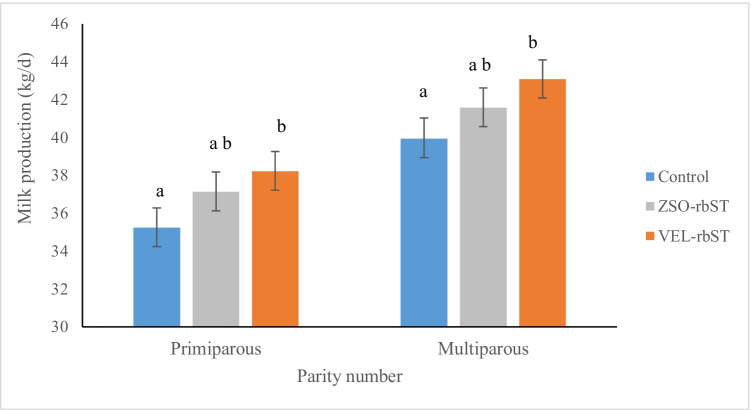
Milk production (kg/day) of primiparous and multiparous cows treated with rbST (VEL-rbST or ZSO-rbST) or untreated (control) cows during the entire 168 days of study. Average milk production within each group by parity with different letters (a–b) are significantly different (*p* < 0.05)

A significant effect of parity was observed on milk production (*p* < 0.01) as well as between treatments (*p* < 0.05). Significant differences were also observed between the group of cows from the control group and the cows with VEL-rbST (*p* < 0.05) but not between VEL-rbST and ZSO-rbST (*p* > 0.05) in both primiparous and multiparous cows (Fig. 1). In primiparous cows, the average milk production in the control group, ZSO-rbST, and VEL-rbST was 35.2, 37.1, and 38.2 kg/day while in multiparous cows, it was 39.9, 41.6, and 43.1 kg/, respectively. For primiparous cows, the difference in milk production between the VEL-rbST and control groups was 3 kg/day while in multiparous cows, it was 3.2 kg/day for VEL-rbST. This additional milk production could represent the economic benefit of the use of VEL-rbST for the farmer. The additional economic income due to the effect of the application of the rbST formulations is presented in Table 2. In primiparous cows, the application of ZSO-rbST and VEL-rbST had an additional increase of US $0.13 and US $0.58 per day, respectively, while in multiparous cows, this increase was US $0.05 and US $0.66 per day for ZSO-rbST and VEL-rbST, respectively. The calculations consider the average additional milk production per rbST (kg/cow/day), cost of rbST ($/cow/day), and price of milk ($/kg).

**Table 2:** Economic response by ZSO-rbST and VEL-rbST application in primiparous and multiparous cows

	Primiparous	Primiparous	Multiparous	Multiparous
	ZSO-rbST	VEL-rbST	ZSO-rbST	VEL-rbST
Average additional milk production (kg/cow/day)	1.9	3.0	1.7	3.2
Cost of rbST ($/cow/day)	0.65	0.65	0.65	0.65
Milk price ($/kg)	0.41	0.41	0.41	0.41
Income for additional milk production ($/cow/day)	0.78	1.23	0.70	1.31
Income due to rbST application ($)	0.13	0.58	0.05	0.66

### Effect of rbST on milk production in the 12 injection cycles

**Fig. 2:**
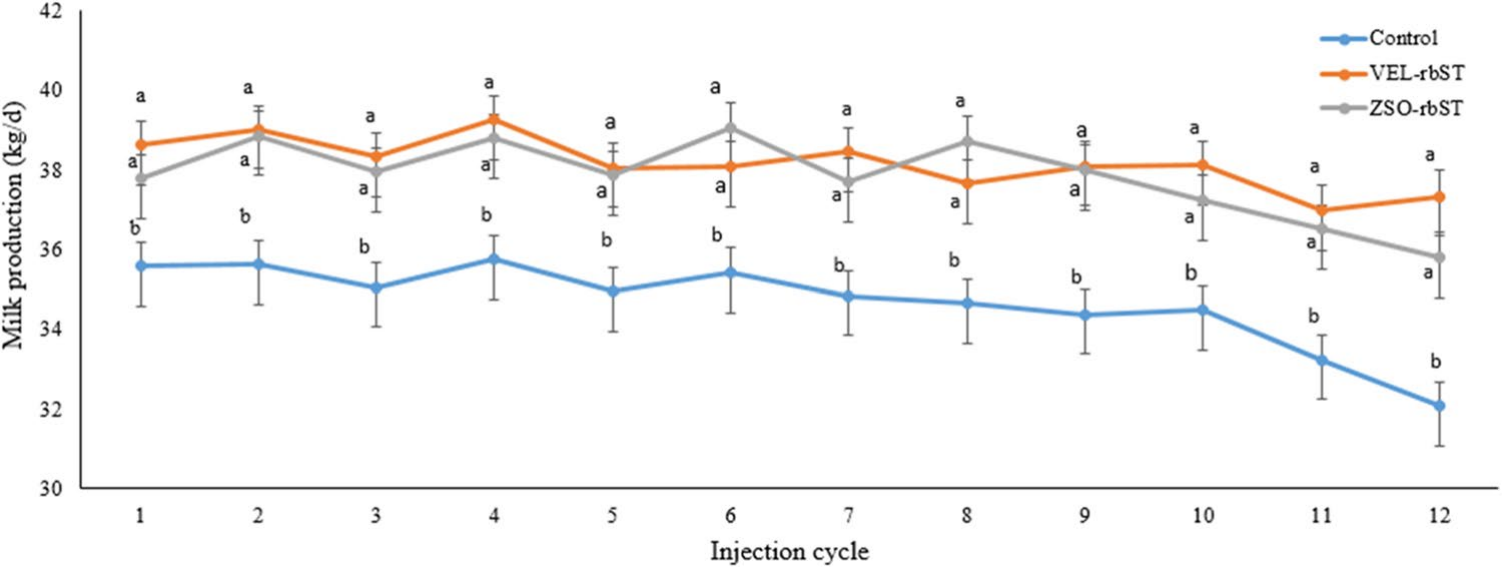
Milk production (kg/day) of primiparous cows treated with rbST (VEL-rbST or ZSO-rbST) or untreated cows (control) during the 12 injection cycles. Average milk production within a cycle with different letters (a–b) are significantly different (*p* < 0.05)

**Fig. 3:**
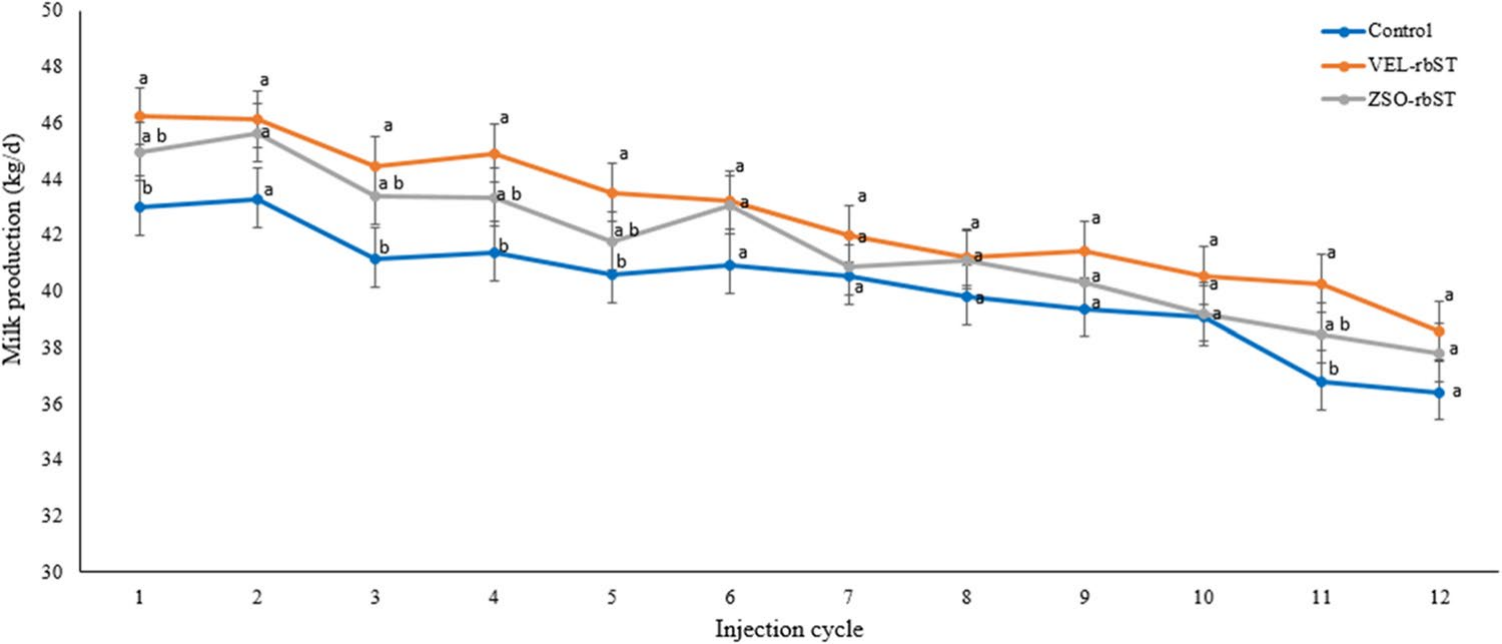
Milk production (kg/day) of multiparous cows treated with rbST (VEL-rbST or ZSO-rbST) or untreated cows (control) during the 12 injection cycles. Average milk production within a cycle with different letters (a–b) are significantly different (*p* < 0.05)

In primiparous cows, differences in milk production were found in the 12 cycles between cows treated with rbST and the control (*p* < 0.05) but not between rbST formulations (*p* > 0.05) (Fig. 2), while in multiparous cows, no differences were found between ZSO-rbST and the control (*p* > 0.05), or between rbST formulations (*p* > 0.05) but differences were found between multiparous cows treated with VEL-rbST and the control (*p* < 0.05) in injection cycles 1, 3, 4, 5, and 11 (Fig. 3). In primiparous cows, the average milk production in the control, ZSO-rbST, and VEL-rbST cows was 34.7, 37.9, and 38.2 kg/day while in multiparous cows, they were 40.2, 41.6, and 42.7 kg/day respectively.

### Effect of rbST on milk production within the injection cycle

**Fig. 4:**
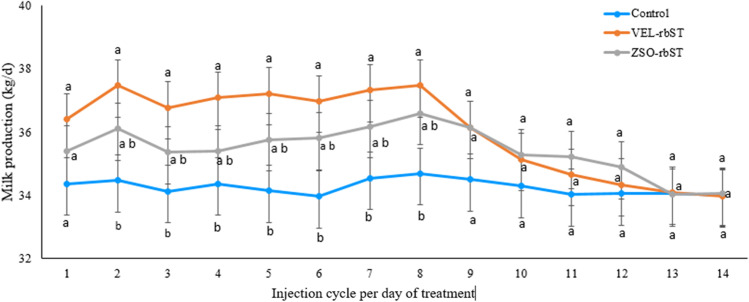
Milk production (kg/day) of primiparous cows treated with rbST (VEL-rbST or ZSO-rbST) or untreated (control) cows in the 14 days of the 12 injection cycles. Average milk production within a cycle with different letters (a–b) are significantly different (*p* < 0.05)

**Fig. 5:**
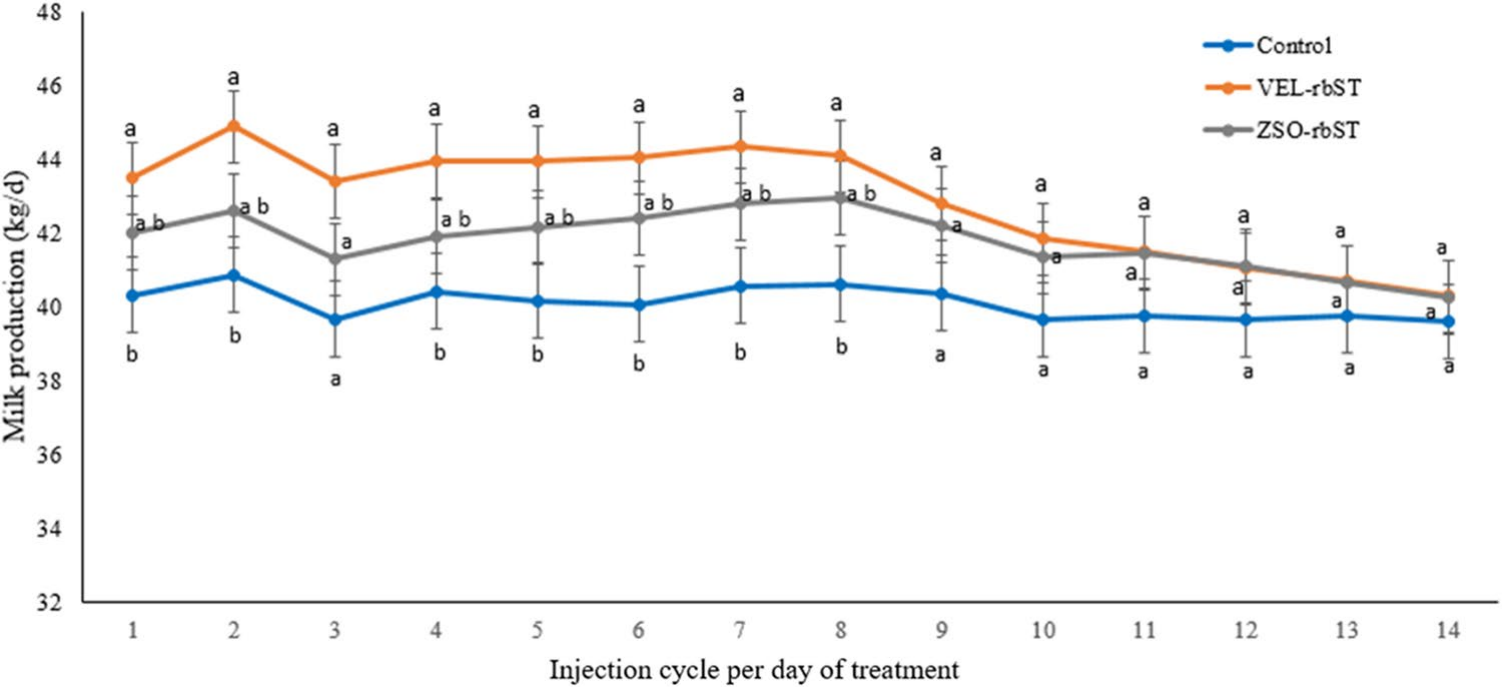
Milk production (kg/day) of multiparous cows treated with rbST (VEL-rbST or ZSO-rbST) or untreated cows (control) in the 14 days of the 12 injection cycles. Average milk production within a cycle with different letters (a–b) are significantly different (*p* < 0.05)

In primiparous and multiparous cows, no significant differences were observed in milk production in the 14 days of the cycle between the rbST evaluated cows (*p* > 0.05). The milk production of primiparous and multiparous cows treated with ZSO-rbST was not higher than that of control cows (*p* > 0.05) in each 14 days cycle. However, in primiparous cows, differences were found between cows treated with VEL-rbST and the control (*p* < 0.05) on days 2, 3, 4, 5, 6, 7, and 8 (Fig. 4). In multiparous cows, differences were found between cows treated with VEL-rbST and the control (*p* < 0.05) on days 1, 2, 4, 5, 6, 7, and 8 (Fig. 5). In primiparous cows, the average milk production of control, ZSO-rbST, and VEL-rbST cows was 34.3, 35.4, and 36.1 kg/day while in multiparous cows, it was 40.1 41.8 and 42.9 kg/day respectively. Primiparous cows with VEL-rbST or ZSO-rbST produced 1.8 and 1.2 kg/day more than control cows, respectively, while multiparous cows produced 2.8 and 1.7 kg/day more than the control, respectively.

### Effect of rbST on body condition

According to the current investigation, no significant effect of parity number on body condition (*p* > 0.05) was observed. The average body conditions in VEL-rbST, ZSO-rbST, and control-treated cows were 3.05, 3.05, and 3.09 in the 12 injection cycles and were not different (*p* > 0.05).

## Discussion

The increase in milk production observed in primiparous and multiparous cows of the VEL-rbST group with respect to the control is within the range of an increase of 3–5 kg/day reported by other researchers (Chalupa et al. 1988; Huber et al. 1997; St-Pierre et al. 2014). As indicated by Bauman (1999), the use of rbST induces an increase in the blood concentration of growth hormone and insulin-like growth factor (IGF-1) which improves milk production mainly through greater lipolysis and prioritization of nutrients toward the mammary gland. However, a higher effect on milk production is observed in multiparous than primiparous cows treated with rbST which was evidenced by previous studies (Leornard et al. 1990; Posada et al. 2008; Mellado et al. 2011; De Morais et al. 2017). The explanation is that even though primiparous cows could have more endogenous bST than multiparous cows, during the first lactation cows continue growing and the nutrients are distributed to cover growth requirements and milk production (McBride et al. 1988; Wathes et al. 2007). In this sense, the growth stage of primiparous cows would reduce the increases in milk production induced by rbST. In primiparous and multiparous cows, no significant differences were found between evaluated rbST, which is similar to that indicated by Posada et al. (2008), Barrios (2015), and Flores et al. (2019). On the other hand, Almeida and Viechnieski (2011) reported significant differences between rbST, where cows injected with VEL-rbST produced 1.6 kg/day more milk than those treated with ZSO-rbST, while De Morais et al. (2017) reported differences between ZSO-rbST and VEL-rbST, being higher for primiparous (+ 2 kg/day) and multiparous cows (+ 1.3 kg/day) treated with ZSO-rbST. These two results are different from those obtained in the present study and may be attributable to differences in general management conditions that are difficult to detect, which is one of the main sources of variation in the response of dairy cows to rbST (Shibru, 2016). Although there was no difference, the VEL-rbST treatment was numerically superior during most of the experimental period to the ZSO-rbST treatment. This is reflected for the present study in the additional increase in income promoted by the VEL-rbST treatment in primiparous and multiparous cows ($0.58 and $0.66, respectively). Similarly, Posada et al. (2008) performed in Colombia a micro economic analysis in order to establish a benefit/cost ratio, reporting that the rbST-treated group exhibited the best ratio. As indicated also by Tauer (2016) over the 20 years that this technology has been used in the USA, annual decreases in the cost of producing milk demonstrate that rbST is a cost-reducing technology.

The evaluation of the rbST response in the 12 injection cycles showed that milk production of primiparous cows with both of the rbST formulations was different from the control in all cycles while in multiparous ZSO-rbST it was not different from the control in any cycle and VEL-rbST was superior in 5 of the 12 cycles. When comparing these results with similar studies, a variable response is found. In the case of primiparous cows, the results are similar to those reported by Barrios (2015) but differ from the data of De Morais et al. (2017) who indicate a higher milk production for cows with ZSO-rbST than with VEL-rbST in 6 of 17 injection cycles. On the other hand, in multiparas, the results of this study agree with those of Almeida and Viechnieski (2011) who reported that milk production of multiparous cows with VEL-rbST was statistically higher than other commercial rbST in all the cycles evaluated but differ from De Morais et al. (2017) who reported that the milk production of cows with rbST was higher in cows with ZSO-rbST. From the present study, it is observed that the milk production response to rbST of primiparous cows is more evident than for multiparous cows but the nature of this study does not explain that difference in response.

In relation to the evaluation of the rbST response on milk production in the 14 days of the 12 injection cycles, rbST formulations were not different in primiparous or multiparas, unlike De Morais et al. (2017) who report that in primiparous the group with ZSO-rbST was greater than VEL-rbST in 9 days of the cycle, while in multiparous primiparous, it was greater in 8 days. In the present study, it is observed that the difference in production was observed in the first half of the application cycle in relation to what was obtained in the rest of the days. This is similar to what was found by De Morais et al. (2017), who detailed that the highest production of the treatment with ZSO-rbST was concentrated between days 3 and 9 of the injection cycle and for VEL-rbST, between days 2 and 8. Figures 4 and 5 show that differences were found between primiparous cows treated with VEL-rbST and the control on days 2, 3, 4, 5, 6, 7, and 8, while in multiparous cows, the differences were on days 1, 2, 4, 5, 6, 7, and 8. For days 9 to 14, there was no significant difference between treatments which is consistent with what was observed by Bauman et al. (1989) who reported peak production at day 7 of the cycle.

There are numerous studies that show increases in IGF-1 production explain the improvement of milk production in cows treated with various formulations of rbST (Schams, 1989; Vicini et al. 1991; Azizan et al. 1994; Collier et al. 2008; Castigliego et al. 2009) without definition if differences are related to the rbST vehicle. In our study, the results of milk production could indicate that the impact of the vehicle on the release profile of rbST from VEL-rbST could be better than ZSO-rbST due to the higher milk production in 8 of the 14 days of the injection cycle in both primiparous and multiparous cows.

The rbST formulations had no significant effect on the body condition of the cows as reported by Posada et al. (2008) and De Morais et al. (2017). Unlike other studies where body condition is reduced (Remond et al. 1991, Thomas et al. 1991 and Stehr et al. 2001), in our study, the results could be due to the fact that the animals started the study with more than 10 weeks of lactation on average exceeding the first 8 weeks postpartum which are critical for negative energy balance. Another possible explanation of the maintenance of body condition in our study may be due to the increase in voluntary feed consumption, which although not measured during the test, different studies have reported an increase in feed consumption in animals that were administered rbST (Dohoo et al. 2003; St-Pierre et al. 2014). This in turn serves to cover the requirements of the additional milk production without compromising body condition.

## Conclusions

Application of the VEL-rbST formulation to lactating Holstein cows for a period of 168 days increased milk production in primiparous and multiparous cows by 3.0 and 3.2 kg/day respectively, compared to noninjected cows. No differences in milk production were found between the ZSO-rbST and control groups or between the two rbST formulations evaluated. When the effect of rbST per injection cycle of 14 days was analyzed, in primiparous cows, differences in milk production were found in the 12 cycles between the two rbST formulations in comparison with control cows, while in multiparous cows, differences were found between VEL-rbST and the control in 41% of the cycles but not between ZSO-rbST and the control for both primiparous and multiparous cows. No significant differences were found in the body condition of the cows between the two rbST formulations and the control group. Although no significant differences were found on milk production between the two formulations, VEL-rbST promoted higher milk production than ZOS-rbST, which had a positive impact on the economic income of the farmer and could be an alternative to improve the profitability of the dairy business in tropical conditions.

## Data Availability

All authors ensure that all data and materials support the findings and comply with field standards. This work was conducted in Agropecuaria Doña Francisca which gave informed consent to data collection from their farm and animals.
